# A *Promotores de Salud* Intervention to Reduce Cardiovascular Disease Risk in a High-Risk Hispanic Border Population, 2005-2008

**Published:** 2010-02-15

**Authors:** Héctor G. Balcázar, Hendrik de Heer, Lee Rosenthal, Maria O. Duarte, Melissa Aguirre, Leticia Flores, Flor A. Puentes, Melchor Ortiz, Victor M. Cardenas, Leslie O. Schulz

**Affiliations:** University of Texas at Houston Health Science Center, School of Public Health, El Paso Regional Campus; University of Texas at El Paso, El Paso, Texas; University of Texas at El Paso, El Paso, Texas; University of Texas at El Paso, El Paso, Texas; Centro San Vicente, El Paso, Texas; El Paso Community College, El Paso, Texas; Texas Department of State Health Services, El Paso, Texas; University of Texas at Houston Health Science Center, El Paso Regional Campus, El Paso, Texas; University of Texas at Houston Health Science Center, El Paso Regional Campus, El Paso, Texas; Northern Arizona University, Flagstaff, Arizona

## Abstract

**Introduction:**

The high prevalence of cardiovascular disease (CVD) in the Hispanic population of the United States, together with low rates of health insurance coverage, suggest a potential cardiovascular health crisis. The objective of Project HEART (Health Education Awareness Research Team) was to promote behavior changes to decrease CVD risk factors in a high-risk Hispanic border population.

**Methods:**

Project HEART took place from 2005 through 2008 as a randomized community trial with a community-based participatory research framework using *promotores de salud* (community health workers). A total of 328 participants with at least 1 CVD risk factor were selected by randomizing 10 US Census tracts in El Paso, Texas, to either the experimental or the control group. The experimental group (n = 192) was assigned to a series of 8 health classes using the *Su Corazón, Su Vida* curriculum. After 2 months of educational sessions, the group was followed for 2 months. The control group (n = 136) was given basic educational materials at baseline, and no other intervention was used. Main outcomes of interest included changes in health behaviors and clinical measures.

**Results:**

Participants in the experimental group showed more awareness of CVD risk factors, more confidence in the control of these factors, and improved dietary habits (ie, lower salt and cholesterol intake, better weight-control practices) compared with the control group. Total cholesterol was 3% lower in the experimental than in the control participants, and non–high-density lipoprotein cholesterol and low-density lipoprotein cholesterol were both 5% lower.

**Conclusion:**

The HEART trial suggests that community health education using *promotores de salud* is a viable strategy for CVD risk reduction in a Hispanic border community.

## Introduction

Heart disease and stroke are the leading causes of death among Hispanics, including those of Mexican origin (1,2). The "Hispanic paradox" is that morbidity and mortality for chronic diseases such as cardiovascular disease (CVD) are lower than for other racial and ethnic groups, despite the low socioeconomic status of the Hispanic population (3). Evidence against this paradox, however, has been growing. Some epidemiologic reports suggest that CVD mortality for Hispanics, the largest and fastest-growing minority population in the United States (4), are equal to and in some instances higher than for non-Hispanic whites (5,6).

The high prevalence of CVD risk factors among Hispanics, together with the lack of health insurance coverage for screening and limited public health capacity for prevention and control, suggests a looming Hispanic cardiovascular health crisis (7). The community outreach model of public health using community health workers (*promotores*) has been proposed as a viable approach to reduce heart disease and stroke among Hispanics (8,9). A systematic review of US-based randomized controlled trials using *promotores* supports the use of this model for addressing CVD and its risk factors (10).

Few randomized controlled trials have studied the use of *promotores* to reduce CVD risk factors in the Hispanic border community. Two interventions used have shown how a *promotora* model can be integrated into community-based chronic disease prevention to address CVD in Hispanics (11,12). *Secretos de la Buena Vida* (Secrets of the Good Life) was designed to improve dietary and nutritional habits among Spanish-speaking Hispanics/Latinos in California, and *Pasos Adelante* (Steps Forward) adapted the *Su Corazón, Su Vida* (Your Heart, Your Life) curriculum of the *Salud Para Su Corazón* (Health for Your Heart) program (13) to reduce risk factors for CVD, diabetes, and other chronic diseases among Arizona Hispanics. *Salud Para Su Corazón* is an initiative of the National Heart, Lung, and Blood Institute based on the model of community-based participatory research (CBPR) and has been used in other *promotores* interventions for CVD and its risk factors (13-16).

This article describes Project HEART (Health Education Awareness Research Team), which was based on *Salud Para Su Corazón* and used a CBPR approach to support the *promotora* model. The HEART intervention promoted a series of positive changes in behaviors to increase awareness of the need to reduce clinical risk factors such as high cholesterol and high blood pressure among Hispanic participants in a large metropolitan border population.

## Methods

### The CBPR agenda for HEART

Project HEART was the first phase (2005-2008) of a CBPR initiative and included 1) 3 academic partners: the University of Texas at El Paso, the University of Texas at Houston Health Science Center School of Public Health, El Paso Regional Campus, and El Paso Community College; 2) a community clinic, Centro San Vicente, that provides services to residents of El Paso who do not have adequate health insurance coverage; 3) *promotores* from a network of partner organizations in El Paso; and 4) a community advisory council. Approval was obtained from both institutional review boards of the University of Texas at El Paso and the University of Texas at Houston Health Science Center School of Public Health.

During the first year of the project (September 2005-August 2006), a variety of CBPR strategies were used for Project HEART to engage different constituencies and partners in a dialogue about how to decrease the prevalence of CVD risk factors in Hispanics (most of Mexican origin) who live in El Paso, Texas. They included a *promotora* community forum, focus groups, and a community advisory council. Information about these activities can be obtained from the first author (HB).

### Development of *promotora* training

*Promotores* from the network of partner organizations were trained for 1 week with the *Su Corazón, Su Vida* curriculum. This curriculum has been used in several of the *Salud Para Su Corazón* *promotora* community interventions (13-16). Twenty *promotores* participated in approximately 16 to 18 hours of training to complete *Su Corazón, Su Vida* lessons taught by a lead *promotora* from Centro San Vicente clinic. From the 20 *promotores*, 3 were hired by the clinic to participate in the 4-month project.

### Recruitment

Recruitment of participants for the 4-month intervention started in the fall of 2006. Ten US Census tracts in the 79915 zip code (area of the Lower Valley of El Paso selected by the community advisory council) were randomly assigned to either the experimental or the control group. The Lower Valley area is characterized by a larger percentage of residents of Hispanic descent (94%) and lower mean educational attainment than the rest of El Paso. Only approximately 5% of residents have an education beyond high school, the median annual family income is approximately $26,000, and approximately 26% of families live below the federal poverty threshold (17).

Blocks within each census tract were assigned to recruiters (graduate students and employees of Centro San Vicente clinic), who were unaware whether the census tract was assigned to the experimental or control group. Using a screening instrument designed for the project (Appendix), recruiters visited 3,959 households to determine whether their inhabitants qualified for the study. To qualify for the study, participants had to be aged 30 to 75 years and have at least 1 self-reported risk factor for CVD (smoking, overweight or obese, diabetes, hypertension, or high cholesterol). Exclusion criteria were pregnancy, having a history of CVD, or not planning to stay in El Paso for the remainder of the study. For people who met the inclusion criteria, the study was explained and informed consent was sought. Only 1 participant per household was selected. The intervention phase took place from the fall of 2006 through the spring of 2007.

### Intervention design

The experimental group was assigned to a series of 8 health classes using the *Su Corazón, Su Vida* curriculum conducted by *promotores*. Each class lasted approximately 2 hours and was delivered every week for 2 months. Participants were then followed for 2 months. Follow-up consisted of 3 telephone calls and a small group session at Centro San Vicente clinic guided by the *promotores* to discuss changes made as a result of the *Su Corazón, Su Vida* classes and to encourage further changes. The control participants were given the basic educational materials from the curriculum in person at the time of the baseline assessment. No *promotora* involvement was provided to the control group.

### Baseline and postintervention assessments

Height, weight, waist circumference, blood pressure, hemoglobin A1c (HbA1c), and lipids were measured for all participants at baseline and 4 months after the intervention. Body mass index, metabolic syndrome, and Framingham 10-year CVD risk factor scores were also calculated by using standard protocols (18,19). Prehypertension and hypertension prevalence estimates were calculated according to accepted standards from the blood pressure data obtained from participants (20). The questionnaire used validated behavioral measures from previous *Salud Para Su Corazón* *promotora* interventions (13-16). The questions assessed 1) perceived susceptibility (feeling that CVD risk factors put them at risk; 3 questions), 2) perceived severity (feeling that CVD is a serious condition; 4 questions), 3) perceived benefits (benefits of behaviors that will help them control CVD risk factors; 7 questions), 4) self-efficacy (confidence in their ability to perform certain behaviors to control CVD risk factors; 6 questions). Responses were measured on a Likert-type scale of 1 to 4 (strongly disagree to strongly agree or not at all confident to very confident for self-efficacy). Demographic characteristics and medication use also were assessed.

Finally, the My Habits Scale previously tested in several *Salud Para Su Corazón* models (13-16) was used to assess participants' heart-healthy behaviors associated with salt consumption (9 items), cholesterol and fat consumption (7 items), and weight control (7 items). These subscales have shown acceptable reliabilities (Cronbach α coefficients>0.70) in similar *promotora* studies (13,15).

### Statistical analyses

Differences between baseline demographic variables for the experimental and control groups were tested by using χ^2^ tests for frequencies or independent-samples *t* test for continuous variables. Unadjusted values for between-group differences before and after the intervention were obtained by using paired-samples *t* tests. Between-groups analysis of covariance was used to evaluate postintervention results, adjusting for baseline values and covariates that were significant between groups at baseline. An intent-to-treat analysis was used for all comparisons.

## Results

Of the 1,395 people who were asked about their willingness to answer the screening questions, 993 (71%) agreed. Of these, 568 were eligible for the study and 407 (71%) agreed to participate. Of the 407 who agreed to participate, 328 (81%) were measured at baseline (192 in the experimental group and 136 in the control group) and 284 were measured at follow-up (158 in the experimental group and 126 in the control group), a retention rate of 87%.

### Unadjusted findings

At baseline, more than 70% of participants were female, and the mean age was 54 years (Table 1). More than 90% of participants spoke Spanish or were bilingual, and approximately 40% reported having no health insurance. More than half of the participants were born in Mexico (53%). Participants had lived in the United States an average of 39 years. Almost 50% of both experimental and control groups reported a family history of CVD, and more than 65% reported a family history of diabetes. Place of birth, years lived in the United States, language, educational attainment, and self-reported financial status were different between experimental and control groups at baseline. However, except for blood pressure, the groups did not differ on clinical indicators at baseline (data not shown). Fifty-one percent of participants in the experimental group attended all 8 classes. Sixty percent attended at least 4 classes.

For the experimental group, significant decreases were observed at 4-month follow-up for weight, low-density lipoprotein (LDL) cholesterol, total cholesterol, non–high-density lipoprotein (HDL) cholesterol, systolic blood pressure, and diastolic blood pressure (Table 2). For the control group, significant changes were observed for waist circumference (increased), HbA1c (increased), and systolic and diastolic blood pressure (decreased). For both groups, the risk for metabolic syndrome, based on mean number of risk factors, increased slightly. The 10-year risk for CVD using the Framingham score was 5% lower for both groups. For the clinical indicators that were self-reported or calculated, only diastolic hypertension showed a significant decrease from baseline in the experimental group, whereas significant decreases were observed in systolic and diastolic hypertension in the control group.

### Adjusted findings

Only 1 clinical indicator, diastolic blood pressure, was significantly different between the 2 groups after controlling for baseline values and confounders (Table 3). Significant improvements were seen in self-reported behaviors such as weight-control practices, salt intake, and cholesterol and fat intake for the experimental group. Perceived susceptibility to CVD and perceived benefits of behaviors that will help participants control their CVD risk factors were significantly higher in the experimental group (Table 4).

## Discussion

Project HEART successfully implemented the 4-month intervention in an underserved Hispanic community in the Lower Valley of El Paso, Texas. The 4-month educational intervention using *promotores* showed promising results for community efforts to limit risk factors for CVD in Hispanic residents of a US-Mexico border area.

The improvements in self-reported heart-healthy behaviors seen in the experimental group are comparable to the results of similar interventions that have used the *Su Corazón, Su Vida* curriculum of the *Salud Para Su Corazón* program by employing *promotores* in different settings and for different Hispanic communities (14-16). Similar programs in Arizona and California using *promotores* have demonstrated positive lifestyle and nutrition changes among study participants (11-13,21).

To our knowledge, this is the first randomized community trial using *promotores* in a Hispanic border community to evaluate clinical outcomes for CVD risk factors. Elsewhere we have provided empiric evidence that Hispanic border populations tend to have a family history of CVD and diabetes, low perceived knowledge and self-efficacy, poor dietary habits, and a high prevalence of CVD risk factors (22).

In examining unadjusted and adjusted differences in clinical indicators after the intervention, and taking into consideration the demographic characteristics of the sample participants, several trends emerged. First, some effects, particularly on blood pressure, were seen regardless of the group assignment. However, on the basis of unadjusted results, the experimental group had more risk reduction than the control group for many of the clinical indicators. Second, the unadjusted paired-test values suggest a positive trend for the effects of the *promotora* intervention on program participants exposed to the educational intervention. For example, total cholesterol, non-HDL cholesterol, and LDL cholesterol decreased by 3%, 5%, and 5%, respectively, for the experimental group.

Thus, although participation in the 4-month education component improved nutrition behaviors and awareness of CVD risk factors, both experimental and control groups showed some positive changes in clinical indicators. Several explanations are possible. Changes in dietary intake and behavior may have occurred, but the study was not long enough for the changed behavior to be reflected in differences in clinical indicators between the 2 groups. In addition, self-reported intake and awareness may not be as strongly associated with measured clinical indicators as expected.

This study has several limitations. It was conceptualized as part of a CBPR effort among several community and university partners, and these partners wanted to provide clinical results to all participants regardless of group assignment. Both groups received feedback from the study's principal and co-principal investigators (LS, HB) at baseline, which was a powerful incentive for participants regardless of group assignment. Thus, receiving baseline measurements and interacting with the project staff may have been an intervention in itself.

The study randomized participants into intervention and control groups, and this strategy worked well for age, sex, marital status, income, health insurance status, and family history of CVD. Despite randomization, however, some differences in demographic variables were present at baseline. To be conservative in our estimates, the variables that differed between groups at baseline were included as covariates. As a result, the power of the intervention may have been reduced, limiting the ability to find differences in clinical indicators.

The results of this randomized community trial suggest that community health education using *promotores* is a promising strategy to support the expansion of the community outreach model. Integrating this model in clinical settings may help reduce the prevalence of CVD risk factors in the Hispanic community. We have recently provided several recommendations based on an initiative of *Salud Para Su Corazón* and the Health Resources and Services Administration that we consider important for the implementation of future CBPR studies of CVD (16). These are 1) include support groups to monitor changes in clinical indicators and to encourage maintaining positive lifestyle changes made as a result of the intervention; 2) provide a visual representation of clinical measures, such as a graph, to motivate participants; 3) develop a referral, follow-up, and documentation system in clinical settings and integrate this system with *promotores* (13); 4) extend the intervention and evaluation timelines to at least 12 months; and 5) provide ongoing training to program coordinators and *promotores* to foster sustainability of the intervention by engaging participants in CVD risk reduction activities at the community level. Project HEART Phase 2 (2008-2013) is expanding its reach by using *promotores* in collaboration with parks and recreation departments, local YWCAs, community clinics, and other community partners.

## Figures and Tables

**Table 1:** Characteristics of Participants in Project HEART, El Paso, Texas, 2005-2008

**Characteristic^a^**	Control Group (n = 136)^b^	Experimental Group (n = 192)^b^	*P* Value^c^
**Female sex**	90 (68)	138 (75)	.20
**Age, mean (SD), y**	54.0 (13.2)	53.5 (13.4)	.83
**Birthplace**			
Mexico	61 (48)	112 (60)	.03
United States	66 (52)	75 (40)	
**Years of residence in United States, mean (SD)**	41.6 (18.6)	37.1 (18.7)	.03
**Language spoken for survey**			
English only	8 (6)	4 (2)	.02
Spanish only	35 (26)	76 (40)	
Both English and Spanish	90 (67)	103 (58)	
**Years of educational attainment, mean (SD)**	10.7 (3.3)	9.7 (3.5)	.01
**Employed**	47 (35)	58 (30)	.43
**Self-reported financial status**			
Very well off or well off	19 (14)	12 (6)	.02
Getting by	86 (64)	115 (61)	
Not getting by	30 (22)	61 (32)	
**Annual family income**			
<$10,000	50 (38)	71 (38)	.91
$10,000 to <$20,000	48 (35)	68 (37)	
≥$20,000	37 (27)	47 (25)	
**No health insurance**	52 (39)	84 (44)	.28
**Receive food stamps/welfare**	30 (22)	58 (30)	.09
**Family history of CVD**	59 (45)	91 (51)	.34
**Family history of diabetes**	87 (67)	121 (67)	.52
**Marital status**			
Married/living with a partner	92 (68)	119 (63)	.47
Widowed/separated/divorced	33 (24)	48 (26)	
Never married	10 (7)	21 (11)	
**No. of people in household, mean (SD)**	3.3 (1.6)	3.4 (1.8)	.53

Abbreviations: HEART, Health Education Awareness Research Team; SD, standard deviation; CVD, cardiovascular disease.

a All values are reported as no. (%) unless otherwise indicated.

b Values for each question may not correspond to the group total because of missing responses. Percentages may not total 100 because of rounding.

c *P* values were calculating by using χ^2^ test (frequencies) or independent-samples *t* tests (continuous). Frequencies may not add up to group total for experimental or control group because of missing responses.

**Table 2:** Unadjusted Differences From Baseline to Follow-Up for Cardiovascular Disease Clinical Indicators, Project HEART, El Paso, Texas, 2005-2008

Clinical Indicator	Control Group (n = 136)		*P* Value^a^	Experimental Group (n = 192)		*P* Value^a^
	
	Baseline	4-Month Follow-Up		Baseline	4-Month Follow-Up	
**Mean (SD)**						
Body mass index, kg/m^2^	31.1 (6.4)	31.2 (6.5)	.28	31.7 (6.8)	31.6 (6.7)	.34
Weight, lb	183.1 (42.5)	183.0 (43.0)	.84	181.8 (39.1)	180.5 (40.0)	.01
Waist circumference, in	40.2 (6.0)	41.0 (5.7)	.01	40.6 (5.8)	40.7 (5.9)	.49
Framingham risk score^b^	14.3 (11.9)	9.3 (7.0)	<.001	15.5 (13.2)	10.8 (7.9)	<.001
Metabolic syndrome, mean no. of risk factors	2.4 (1.3)	2.6 (1.3)	.01	2.4 (1.2)	2.7 (1.2)	<.001
LDL cholesterol, mg/dL	120.2 (31.9)	119.8 (33.6)	.86	127.6 (36.2)	121.3 (38.0)	.01
HDL cholesterol, mg/dL	42.6 (10.7)	41.5 (11.2)	.11	40.6 (11.2)	40.6 (10.0)	.97
Total cholesterol, mg/dL	190.5 (38.5)	190.5 (42.4)	.99	197.5 (48.5)	192.3 (54.5)	.03
Non-HDL cholesterol, mg/dL	147.9 (37.7)	148.9 (42.9)	.69	155.1 (41.5)	149.3 (43.1)	.01
Triglyceride level, mg/dL	139.1 (82.8)	139.2 (91.7)	.98	134.7 (71.5)	140.9 (77.5)	.21
Fasting blood glucose, mg/dL	95.3 (31.7)	99.8 (40.7)	.09	101.5 (40.3)	105.2 (44.3)	.10
HbA1c, %	6.3 (1.3)	6.5 (1.3)	.01	6.6 (1.5)	6.6 (1.5)	.83
Systolic blood pressure, mm Hg	141.4 (20.5)	132.6 (17.5)	<.001	137.2 (21.8)^c^	131.9 (19.2)	<.001
Diastolic blood pressure, mm Hg	89.4 (16.3)	78.3 (10.8)	<.001	80.0 (10.9)^c^	77.6 (9.4)	.01
**Prevalence, %**						
Smoking	30	21	.09	27	19	.07
Diabetes^d^	30	34	.50	35	40	.32
Systolic hypertension^d^	45	29	.01	37	29	.11
Systolic prehypertension^d^	40	45	.40	36	41	.32
Diastolic hypertension^d^	41	16	<.001	21	10	.01
Diastolic prehypertension^d^	28	24	.46	28	25	.55
Taking hypertension medication^e^	41	40	.78	42	45	.59
Taking lipid-lowering medication^e^	27	29	.63	29	35	.28
Taking diabetes medication^e^	26	22	.52	29	30	.79

Abbreviations: HEART, Health Education Awareness Research Team; SD, standard deviation; LDL, low-density lipoprotein; HDL, high-density lipoprotein; HbA1c, hemoglobin A1c.

a *P* values were calculated by using paired-samples *t* tests.

b Framingham score based on age, LDL cholesterol, HDL cholesterol, smoking status, diabetes diagnosis (self-report, HbA1c >7%, or fasting glucose >126 mg/dL).

c Significantly different from control at baseline, *P* < .05.

d Diabetes defined as HbA1c >7%, fasting blood glucose >126 mg/dL, or self-report; systolic hypertension defined as >140 mm Hg and prehypertension defined as >120 mm Hg; diastolic hypertension defined as >90 mm Hg and prehypertension defined as >80 mm Hg.

e All variables regarding medication intake are self-reported.

**Table 3:** Adjusted Postintervention Differences at Follow-Up for Cardiovascular Disease Clinical Indicators, Project HEART, El Paso, Texas, 2005-2008

Clinical Indicator	Control Group 4-Month Follow-Up^a^ (n = 126)	Experimental Group 4-Month Follow-Up^a^ (n = 158)	*P* Value^b^
Body mass index, kg/m^2^	31.3 (6.7)	31.1 (6.3)	.28
Weight, lb	181.1 (44.2)	180.5 (37.5)	.44
Waist circumference, in	41.0 (5.9)	40.4 (5.7)	.09
Framingham risk score^c^	9.5 (6.7)	10.4 (7.8)	.26
Metabolic syndrome, mean no. of risk factors	2.6 (1.2)	2.7 (1.2)	.32
LDL cholesterol, mg/dL	123.0 (33.8)	118.6 (37.8)	.20
HDL cholesterol, mg/dL	41.5 (11.2)	41.6 (10.2)	.98
Total cholesterol, mg/dL	195.2 (42.7)	189.5 (54.5)	.16
Non-HDL cholesterol, mg/dL	152.4 (43.4)	146.3 (42.3)	.10
Triglyceride level, mg/dL	139.3 (94.5)	143.6 (78.6)	.64
Fasting blood glucose, mg/dL	102.7 (42.2)	101.9 (39.8)	.80
HbA1c, %	6.6 (1.4)	6.5 (1.4)	.09
Systolic blood pressure, mm Hg	130.5 (16.7)	132.6 (19.4)	.20
Diastolic blood pressure, mm Hg	75.5 (10.6)	79.8 (9.3)	<.001

Abbreviations: HEART, Health Education Awareness Research Team; LDL, low-density lipoprotein; HDL, high-density lipoprotein; HbA1c, hemoglobin A1c.

a Parenthetical values represent the standard deviations.

b Between-groups analysis of covariance for the postintervention follow-up values were adjusted for baseline value, self-reported birthplace, language chosen for baseline survey, years of residence in United States, educational attainment, and financial status.

c Framingham score based on age, LDL and HDL cholesterol levels, smoking status, diabetes diagnosis (self-report, HbA1c >7%, or fasting blood glucose >126 mg/dL).

**Table 4:** Adjusted Postintervention Differences at Follow-Up for Nutrition-Related Behaviors and Health Beliefs, Project HEART, El Paso, Texas, 2005-2008

Behavior/Belief Indicator	Control Group 4-Month Follow-Up (n = 126)^a^	Experimental Group 4-Month Follow-Up (n = 158)^a^	*P* Value^b^
Weight-control practices^c^	1.9 (0.6)	2.0 (0.6)	.01
Salt intake^c^	1.8 (0.5)	2.0 (0.5)	<.001
Cholesterol and fat intake^c^	1.7 (0.6)	1.9 (0.7)	.01
Perceived severity^d^	3.6 (0.5)	3.6 (0.4)	.33
Perceived benefits^d^	3.6 (0.5)	3.7 (0.4)	.01
Perceived susceptibility^d^	3.4 (0.5)	3.5 (0.4)	.01
Self-efficacy^d^	3.3 (0.5)	3.4 (0.4)	.13

Abbreviation: HEART, Health Education Awareness Research Team.

a Values indicate responses on a Likert-type scale of 1 to 4, followed by the standard deviation in parentheses.

b Between-groups analysis of covariance for the postintervention values were adjusted for baseline value, self-reported birthplace, language chosen for baseline survey, years of residence in United States, educational attainment, and financial status.

c Measured with My Habits Behavioral Self-Reported Scales (13-16) indicating never to always engaging in healthy behaviors.

d Measured with a questionnaire indicating strongly disagree to strongly agree or not at all confident to very confident for self-efficacy.

## Appendix. Screening Questionnaire for Establishing Eligibility, Project HEART (Health Education Awareness Research Team), El Paso, Texas, 2005-2008

1. Do you plan to move out of town in the next 6 months?
  - Yes
  - No
  - Don't know
  - Refused
2. What is your age? 
3. Are you pregnant or plan to become pregnant in the next 6 months?
  - Yes
  - No
  - Don't know
  - Refused
4. Are you Hispanic or Latino?
  - Yes
  - No
  - Don't know
  - Refused
5. Have you ever been told by a doctor that you have any of the following risk factors?
  - High blood pressure
    - Yes
    - No
    - Don't know
  - High cholesterol
    - Yes
    - No
    - Don't know
  - Diabetes
    - Yes
    - No
    - Don't know
  - Overweight
    - Yes
    - No
    - Don't know
6. Do you smoke cigarettes?
  - Yes
  - No
7. Do you have a history of heart disease?
  - Yes
  - No
  - Don't know

***Checklist for eligibility:***

*Answers: 1. No, 2. 35 years or older, 3. No, 4. Yes, 5. and 6. At least one yes, 7. No*

***If all 1-7 answers check with eligibility criteria they are eligible: "You are eligible to participate in the HEART study. I will now read to you the consent form and if you decide to participate, we will schedule an appointment for another interview and blood tests."***
